# Development of the European Society of Hypertension guidelines for the management of arterial hypertension: comparison of the helpfulness of ESH 2013, 2018, and 2023 guidelines

**DOI:** 10.1097/HJH.0000000000003985

**Published:** 2025-02-17

**Authors:** Akos Koller, Zoltán Járai, Johanna Takács

**Affiliations:** a Department of Morphology and Physiology, Faculty of Health Sciences, Semmelweis University; b Research Center for Sports Physiology, Hungarian University of Sports Science; c Department of Translational Medicine, Faculty of Medicine, HUN-REN-SE Cerebrovascular and Neurocognitive Disease Research Group, Semmelweis University, Budapest, Hungary; d Department of Physiology, New York Medical College, Valhalla, New York, USA; e Department of Cardiology, South-Buda Center Hospital St, Imre University Teaching Hospital; f Section of Angiology, Heart and Vascular Center; g Department of Social Sciences, Faculty of Health Sciences, Semmelweis University, Budapest, Hungary

**Keywords:** certainty, clinical practice guidelines, hypertension, mathematical analysis, quality control

## Abstract

**Objective:**

Over the last decade, the European Society of Hypertension (ESH) published several guidelines (GLs) for the Management of Arterial Hypertension (2013, 2018, and 2023). We hypothesized that the GL has been improved because of the publications of new evidence. Thus, we aimed to examine the development of ESH guidelines (ESH GLs) by comparing their helpfulness regarding the diagnosis and treatment of hypertension.

**Methods:**

A novel mathematical analysis was used to compare ESH GLs. Not only the frequency of Classes of Recommendations (CLASS) and the Levels of Evidence (LEVEL) were examined but a newly developed certainty index (CI) was calculated. This CI allows the CLASS and LEVEL to be assessed together, providing a less biased assessment of GLs, than examining the CLASS and LEVEL independently or related to each other.

**Results:**

The number of recommendations showed continuous and significant increases from 2013 (*N* = 110) to 2018 (*N* = 169), and 2023 (*N* = 269). Examining the frequency of CLASS and/or LEVEL led to biased results, showing both improvements and/or worsening comparing years. However, based on the new analysis, a continuous improvement was shown in the percentage of certainty from 2013 to 2023 (2013: 60.5%, 2018: 72.1%, 2023: 75.3%). Accordingly, the CI was also significantly increased from 2013 (CI: 0.21), to 2018 (CI: 0.44), and to 2023 (CI: 0.51).

**Conclusion:**

The analysis shows that compared to previous GLs, the structure of the ESH 2023 GL has been rearranged and simplified. The higher number of Recommendations indicates a continuously accumulating knowledge regarding the mechanisms, clinical findings, and epidemiology of hypertension. Moreover, the ESH 2023 GL shows a higher degree of certainty and CI, corresponding to a higher level of helpfulness of the ESH 2023 GL for healthcare professionals to diagnose, prevent, and treat hypertension.

## Graphical abstract

**Figure:**
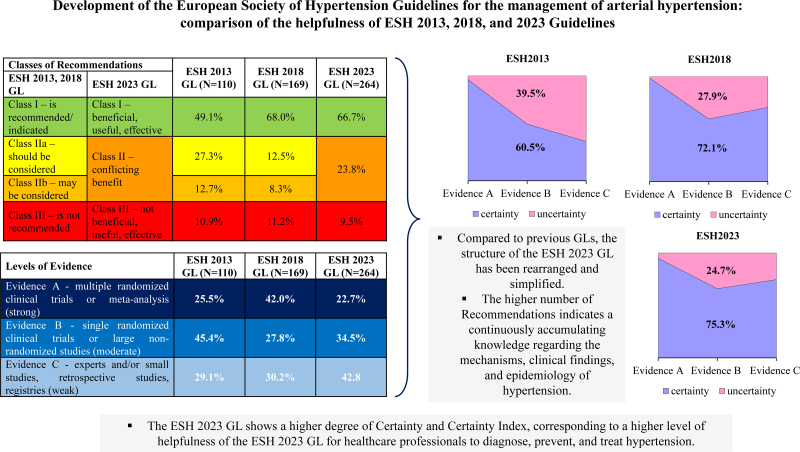

## INTRODUCTION

The European Society of Hypertension (ESH) published several guidelines (GLs) for the management of arterial hypertension in recent years to help physicians and patients make decisions in particular disease conditions. The last one was published in 2023, the ESH 2023 guidelines for the management of arterial hypertension [1].

The year 2023 marks the 20th anniversary of the hypertension guidelines of the ESH, which were published for the first time in 2003. It is evident that guidelines – based on past collected knowledge – are important regarding diagnosis and treatment, to prevent or delay the adverse effects of hypertension.

Several studies conducted in the past few decades have shown that clinical practice guidelines, for the management of cardiovascular diseases, include a small percentage, less than 15%, of the recommendations which are supported by Level A evidence, meaning that multiple randomized clinical trials and meta-analyses providing the highest level of evidence are not present [2–7]. Furthermore, these studies also have demonstrated a steady temporal trend or small increase in Level A evidence [2,4–6]. Based on a recent study comparing 50 clinical practice guidelines from 2011 to 2022, 16% of the recommendations were supported by the highest quality of evidence, Level A, at the same time, there was a slight global increase in Level A in recent years [8].

There are no systematic methods to measure quantitatively the helpfulness or quality of GLs, that is, how much help they provide to the physicians. Moreover, previous assessments/methods also pose several questions. Whether the previous analysis could provide an objective ‘result’ on the helpfulness of a GL, for example, based on: the frequency of recommendations and evidence, the frequency of evidence at each class of recommendation (Level by Class), or vice versa the frequency of recommendations in each level of evidence (Class by Level)? These questions underline the need to further develop the methods of assessing GLs, which can be improved by including implementation advice and translating scientific Evidence into Recommendations.

The ESH 2023 guideline was a further development of the 2018 guideline, in terms of structure and function, covering more than 40 new aspects in detail. In addition, several new clinical trials were completed in recent years, thus we have hypothesized that the 2023 guideline will provide greater help to clinicians in the diagnosis and management of hypertension. We also hypothesized that our method provides an objective assessment of the usefulness of a guideline, as a kind of ‘quality control’, and allows comparison of various medical guidelines.

## METHODS

A comparative mathematical analysis of the ESH GLs (2013, 2018, and 2023) [1,9,10] was conducted based on our previous methods [11,12]. First, the frequency of the Classes of Recommendations and the Levels of Evidence was reported. Then, based on the Levels of Evidence considering each Class of Recommendations the ratio of certainty/uncertainty was calculated. Finally, we formulated a certainty index (CI) (ranging from −1 to +1). A value of −1 implies 100% uncertainty, a value of +1 implies 100% certainty, and a value of 0 denotes 50–50 certainty and uncertainty. For the illustration of the calculation of the CI, see Supplementary File 1.

## RESULTS

### Frequency of the classes of recommendations

The number of Recommendations showed continuous and significant increases from 2013 (*N* = 110) to 2018 (*N* = 169), and 2023 (*N* = 269). From 2013 to 2018, the number of recommendations increased by 53.6%, from 2018 to 2023, it was a 56.2% increase. In sum, ESH 2023 GL included nearly one and a half times more recommendations than ESH 2013 GL.

The frequency of Class I and Class III recommendations showed also an increase from 2013 to 2018, which indicates a higher number of recommendations supporting certain decisions based on Class I: to do and Class III: not to do (ESH 2013 GL: 60%, ESH 2018 GL: 79.2%, ESH 2023 GL: 76.2%). Thus, the rate of recommendations in Class II (IIa,b which were merged to “conflicting benefit” in ESH 2023 GL) decreased to 20% uncertainty (Table 1).

**TABLE 1:**
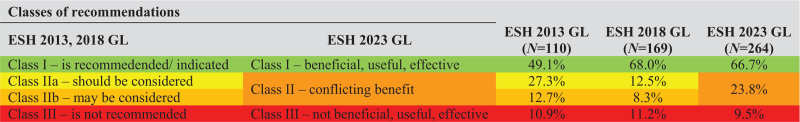
The frequency of the classes of recommendations in the ESH 2013, 2018 and 2023 GLs

### Frequency of the levels of evidence

Based on the frequency data, Level A evidence showed an increase from 2013 to 2018, but a decrease to 2023. The frequency of Level A evidence is one of the lowest in ESH 2023 GL, which raises the question of whether ESH 2023 GL provide less help compared to previous ESH GLs or whether such an analysis based on only frequency data cannot be used to conclude the helpfulness of GLs (Table 2).

**TABLE 2:**
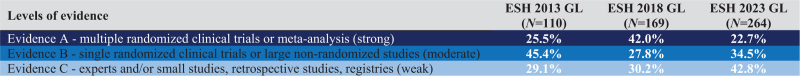
The frequency of the levels of evidence in the ESH 2013, 2018 and 2023 GLs

### The ratio of certainty/uncertainty (%)

**FIGURE 1:**
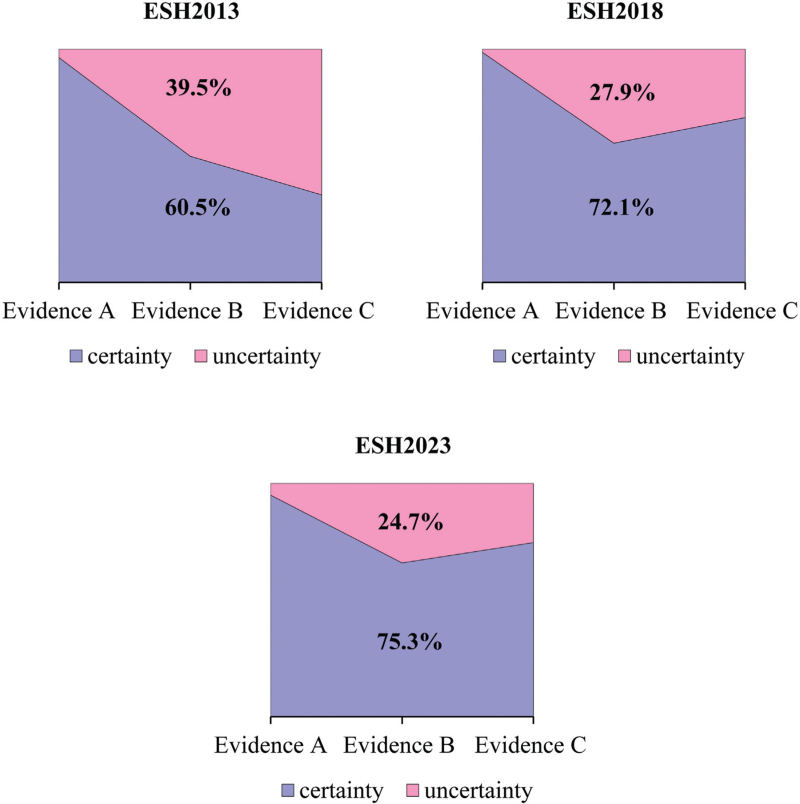
The ratio of certainty/uncertainty in the ESH 2013, 2018 and 2023 GLs.

However, based on the new analysis calculating the ratio of certainty/uncertainty, a continuous improvement was shown in the percentage of certainty from 2013 to 2023 (Fig. 1).

### Certainty index

**FIGURE 2:**
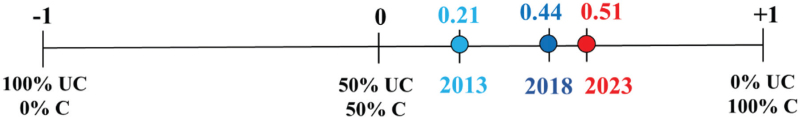
The certainty index in the ESH 2013, 2018, and 2023 GLs.

Accordingly, the CI was also significantly increased from 2013 to 2023, representing the highest, 75.3% of certainty and the lowest, 24.7% of uncertainty in the ESH 2023 GL (Fig. 2).

Finally, using the new analysis, based on the CI, it can be compared various disorders and different GLs. Thus, we examined the CI of hypertension in established cardiovascular disorders and specific settings such as pregnancy, as well as in diabetes mellitus and kidney disease. Then, we demonstrated that the CI is also useful to compare the helpfulness of different GLs.

### The certainty index of recommendations in established cardiovascular disorders and in pregnancy, diabetes mellitus and kidney disease based on the ESH 2023 GL

**FIGURE 3:**
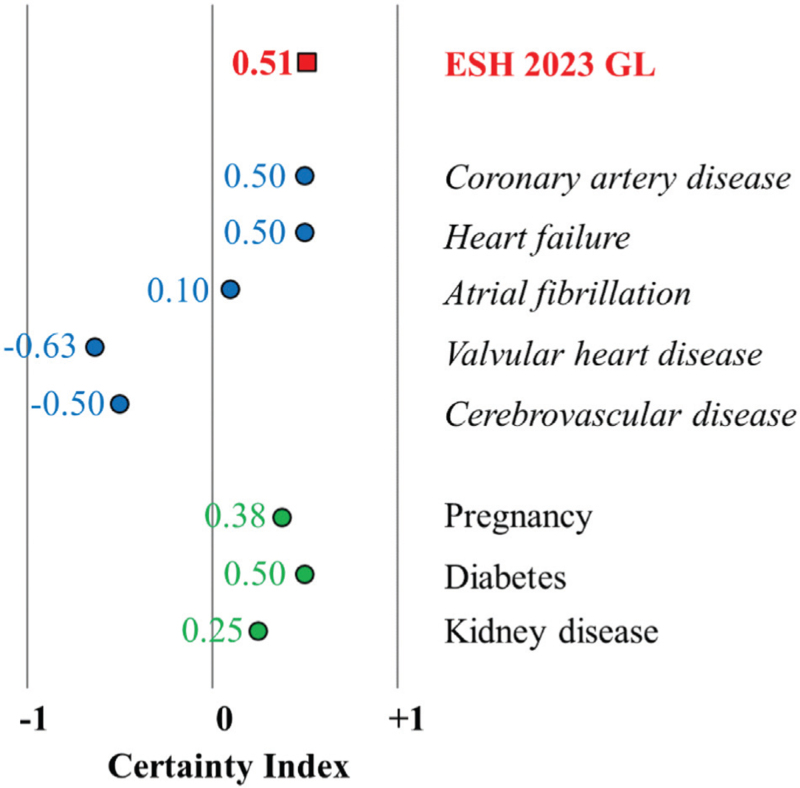
The certainty index of recommendations in established cardiovascular disorders and in pregnancy, diabetes mellitus and kidney disease in the ESH 2023 GL.

Among the examined cardiovascular disorders, valvular heart disease and cerebrovascular disease showed the lowest CI with a certainty of 18.75% and 25%, respectively. The highest CI was found in the case of coronary artery disease and heart failure with a certainty of 75%, such as in diabetes. CI in pregnancy represents a certainty of 68.8%, and in kidney disease, it was 62.5% (Fig. 3).

### The certainty index of different guidelines

To demonstrate the comparison of different GLs, we depicted the calculated CI of three further ESC guidelines: 2018 ESC/EACTS guidelines on myocardial revascularization [13], 2018 ESC guidelines for the management of cardiovascular diseases during pregnancy [14], 2018 ESC guidelines for the diagnosis and management of syncope [15].

**FIGURE 4:**
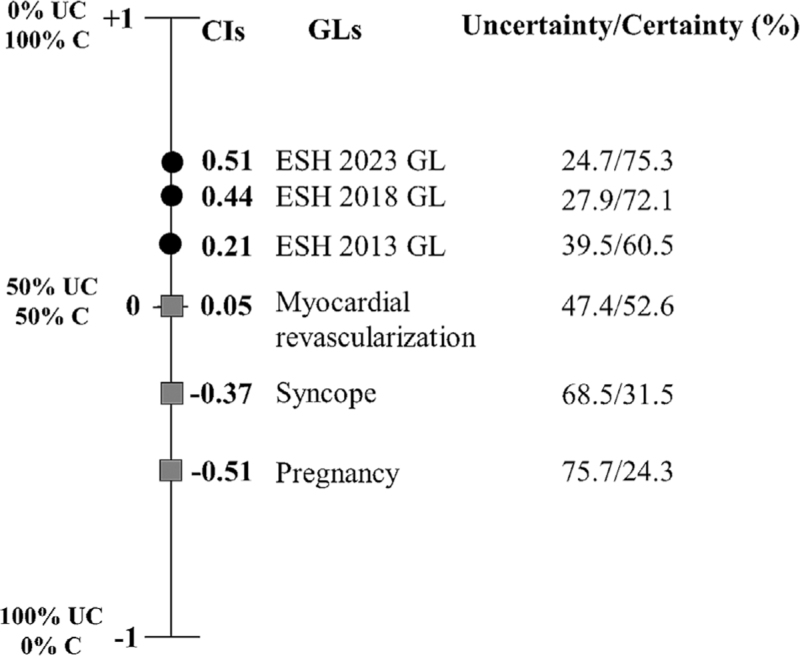
The comparison of the GLs based on the certainty index.

The GL on myocardial revascularization showed a CI of 0.05, which means around 50%–50% of certainty/uncertainty. CIs were negative in syncope and pregnancy GLs, which represents a higher uncertainty than certainty, 68.5% and 75.7%, respectively (Fig. 4).

## DISCUSSION

The mathematical analysis used in this study revealed that there were several changes in the ESH 2023 guideline: the structure has been rearranged, the structure has been simplified, the higher number of Recommendations demonstrates a continuously accumulating knowledge regarding the mechanisms, clinical findings and epidemiology of hypertension, moreover, the ESH 2023 GL shows a higher degree of certainty and CI, corresponding to a higher level of helpfulness of the ESH 2023 GL for healthcare professionals to diagnose and treat hypertension, the method used provides an objective assessment of the usefulness of a guideline, as a kind of ‘quality control’, and allows comparison of various medical guidelines.

Guidelines are an important compass for physicians to make conscious decisions in certain diseased conditions regarding diagnosis and treatments. Thus, they are essential tools, which however as science develops, must be upgraded from time to time.

This is the case for the devastating disease associated with the chronic presence of high blood pressure: hypertension. For that reason, the European Society of Hypertension (ESH) published guidelines (GLs) for the management of arterial hypertension in 2013, 2018 and 2023 [1,9,10], to help physicians and patients make decisions in various hypertensive conditions to prevent or delay the adverse effects of hypertension.

Interestingly, several studies have pointed out that the GLs for the management of cardiovascular diseases include only a small percentage of the recommendations supported by Level A evidence examined over the past few decades [2–8].

However, there are still no existing systematic methods to measure quantitatively the helpfulness or quality of GLs, that is, how much help they provide to physicians. In addition, it is not clear whether previous assessments/methods could provide an objective assessment of the helpfulness of a GL. These are the frequency of Recommendations and Evidence, the frequency of evidence at each Class of Recommendations (Level by Class), or vice versa the frequency of recommendations in each Level of Evidence (Class by Level), which can result in biased frequency data and conclusions. To demonstrate these biased results in the case of the 2013, 2018 and ESH 2023 GL, see Supplementary File 2. Thus, it was necessary to further develop methods for assessing GLs.

The present mathematical analysis clearly showed that the ESH 2023 guideline improved compared to the ESH 2018 guideline, in terms of structure and function, covering more than 40 new aspects in detail. This is likely due to the several new clinical trials that were completed in recent years, thereby providing greater help to clinicians in the diagnosis and management of hypertension.

Here we list some of the new important recommendations in the ESH 2023 GL:

1. Five new, detailed recommendations (four Class I, one Class II) were made on the method and rules of home blood pressure measurement, and on the role of home measurements in decision-making. There are also five recommendations for ambulatory blood pressure monitoring in diagnostics and care.
2. In the case of lifestyle treatment, the list of recommendations has been expanded to include increased intake of potassium (I/B) and stress reduction via controlled breathing exercise or mindfulness-based exercise/meditation (II/C).
3. A new recommendation has been made for the threshold of blood pressure (SBP ≥130 or DBP ≥80 mmHg) requiring treatment in high-risk patients with a strength of recommendation I/A.
4. A detailed proposal containing a total of four recommendations was made for the safety of frail patients (two Class III, one Class I and one Class II; all of them having a level of Evidence C).
5. In terms of antihypertensive strategy, beta-blockers can enter as treatment (I/C) at any stage of the up-titration phase (I/C) even without compelling indication.
6. The treatment strategy for true resistant hypertension has been renewed and renal denervation has been added as a therapeutic option (II/B). There are 4 new recommendations on renal denervation (two I/C and two II/B).
7. For specific forms of hypertension (isolated systolic, isolated diastolic, nocturnal hypertension, treated white-coat and masked hypertension), a total of 12 new recommendations were made (seven Class I, five Class II).
8. Concerning baroreflex failure and autonomic failure, four Class I and three Class II recommendations were formulated (all of them having a level of Evidence C).
9. A new recommendation has been given for starting therapy at the threshold BP value of 140/90 mmHg in gestational hypertension (I/C for everybody and I/A for pregnant women with pre-existing hypertension).
10. For the prevention of hypertension-related cardiovascular (CV) diseases (coronary artery disease, heart failure, atrial fibrillation, valvular diseases, acute stroke), as well as for the treatment of hypertension complicated by these diseases and other diseases that significantly increase CV risk (diabetes, chronic kidney disease, obesity, OSAS, COPD, autoimmune diseases, gout), there are 94 recommendations, almost half of which are new.
11. A new follow-up strategy was created with ten new recommendations (nine Class I and one Class II).

All in all, the new ESH 2023 GL is a remarkable piece of work of many clinicians and basic scientists, which is advancing the diagnosis and treatment of hypertension and associated diseases.

It is important to note that a detailed analysis of the Recommendations from a clinical point of view was outside the scope of the present study. However, the mathematical methods used in the present study can be used to assess objectively the usefulness of a guideline, providing a ‘quality control’, which also allows comparison of various medical guidelines.

By doing so, one can propose a further improvement of future ESH Hypertension GL by including the following:

1. Realize and include that there is no ‘average’ patient (mean ± SD)
2. Age, gender, race, and location (e.g., altitude, weather, season, temperature)
3. Further individual characteristics (e.g., physical activity, diet, lifestyle, genetics, epigenetics, etc.)
4. Collect more evidence, but only where it is low (basic research, clinical research)
5. Better development of decision-making mechanisms, and use of artificial intelligence
6. Quantification of the past decision of the physician and the synergy medical team.

### ESH 2023 guideline certainty index associated with cerebrovascular disease

Although our initial intention was not to discuss the mathematical findings of ESH 2023 GL from the aspect of physicians specialized in hypertension, the intriguing findings regarding the wide range of certainty index of managing hypertension in various cardiovascular diseases prompted us to do it anyway. Interestingly the lowest certainty index was associated with cerebrovascular disease although it is known that this disorder is generally associated with an elevated arterial blood pressure. We propose that it is likely that the adaptation of cerebral blood flow (CBF) autoregulation [16] for many decades can mask the manifestation of cerebrovascular disease.

Perhaps the earliest observation of cerebrovascular disease in hypertension can be made by ophthalmologists who can visualize the retina microcirculation and observe the typical malformation of arterioles and their network with the novel optical coherence tomography (OCTA) method. Because nowadays this diagnostic option is readily available in most hospitals and clinics perhaps it should be utilized more frequently for the early diagnosis of hypertension and other neural diseases [17] and then provide novel evidence for the next edition of the ESH Hypertension guideline increasing the certainty index for the diagnosis and treatments of cerebrovascular disease in hypertension.

Surprisingly, the ESH 2023 GL does not have specific recommendations for antihypertensive therapy for primary or secondary prevention of stroke. This is because cardiovascular disease was generally referred to when the evidence of the benefit of antihypertensive therapy was discussed. Thus, when the threshold for antihypertensive treatment initiation and the target to be achieved is determined, the benefit for CV diseases, including stroke, is generally discussed. What one can analyse, are the recommendations for the management of elevated BP in acute stroke; however, the relative lack of available evidence prevents making unequivocal recommendations.

For example, in the case of haemorrhagic stroke, it is not clear what the blood pressure target should be, and based on the available RCTs, the question is also influenced by how much time has passed since the onset of symptoms [18–20].

In the case of ischemic stroke, most RCTs and meta-analyses have not demonstrated a clear benefit of early blood pressure reduction. In addition, the studies were extremely heterogeneous, regarding both the patient population and the endpoints [21]. That is why the GL made a ‘pragmatic recommendation’ with a level of evidence B in this setting.

### ESH 2023 guideline certainty index associated with hypertension disorders in pregnancy

Another important clinical issue is hypertension disorders in pregnancy, a special condition, which is difficult to model in experimental animal studies. As one can expect the CI associated with hypertension disorders in pregnancy is not too high, due to the relatively little evidence available, and the complex underlying mechanisms eliciting hypertension in pregnancy. Nevertheless, the CI could be further improved by considering recently discovered novel plasma markers [22–24] and more detailed analyses of blood pressure values and their changes, such as pulse pressure [25]. All of these may provide more evidence and earlier recognition of elevated blood pressure and cardiovascular disease in pregnancy and thus will increase the CI in the new ESH GL on hypertension.

On that note, the next ESH GL on hypertension should have more specific recommendations for women and men, because it has been already recognized that the underlying mechanisms, development, symptoms, prevention and treatments of hypertension have sex differences [26,27].

### ESH 2023 guideline and AHA 2024 guideline on hypertension

In contrast to the ESH 2023 GL, the AHA 2024 GL (American Heart Association) has a separate chapter for hypertension management for primary and secondary stroke prevention [28]. In the AHA 2024 GL, hypertension screening among adults has I/C, <130/80 mmHg target BP level to prevent stroke has I/A, the recommended drug classes (thiazide and thiazide-like diuretics, calcium channel blockers, angiotensin-converting enzyme inhibitors, and angiotensin receptor blockers) for stroke prevention has I/A and the use of combination therapy has also I/A recommendation based on “abundant high-quality RCTs and systematic reviews/meta-analyses” [29–35].

Thus, it seems that discrepancies in the two GLs are due to the therapeutic consideration of ‘acute’ vs. ‘chronic’ conditions elicited by cerebrovascular disease, which should be indicated in the next edition of ESH GL.

In conclusion, the mathematical analysis, used in the present study, showed that compared to previous GLs, the structure of the ESH 2023 GL has been rearranged and simplified. The higher number of Recommendations indicates a continuously accumulating knowledge regarding the mechanisms, clinical findings and epidemiology of hypertension. Moreover, the ESH 2023 GL shows a higher degree of certainty and CI, corresponding to a higher level of helpfulness of the ESH 2023 GL for healthcare professionals to diagnose, prevent, and treat hypertension. Nevertheless, further mechanistic, basic science and clinical investigations may further increase the evidence basis of the next ESH GL on hypertension.

## ACKNOWLEDGEMENTS

Conflicts of interest and source of funding: This research was funded by the Ministry for Innovation and Technology Hungary, National Research, Development and Innovation Fund, TKP2020-NKA-17 (A.K.), TKP2021-EGA-37 (A.K.), TKP2021-EGA-25 (J.T.), OTKA K 132596 (A.K.); Hungarian Hypertension Society, HHS-2024 Research Grant; Hungarian Academy of Sciences Post-Covid 2021-34 (A.K. and J.T.), New National Excellence Program of the Ministry for Innovation and Technology from the source of the National Research, Development and Innovation Fund, ÚNKP-23-4-II-SE-30, EKÖP-2024-151 (J.T.).

### Conflicts of interest

There are no conflicts of interest.

## Supplementary Material

Supplemental Digital Content

## Supplementary Material

Supplemental Digital Content
