# Supplementary material for: Development of the European Society of Hypertension guidelines for the management of arterial hypertension: comparison of the helpfulness of ESH 2013, 2018, and 2023 guidelines
Source: J Hypertens. 2025 Feb 17;43(5):852–8. doi: 10.1097/HJH.0000000000003985 (PMC11970597; doi:10.1097/HJH.0000000000003985)
Supplement: Supplemental Digital Content [file jhype-43-852-s001.docx]

**Supplementary File 1**. Forming the Certainty Index (Koller, Takács 2024)

**
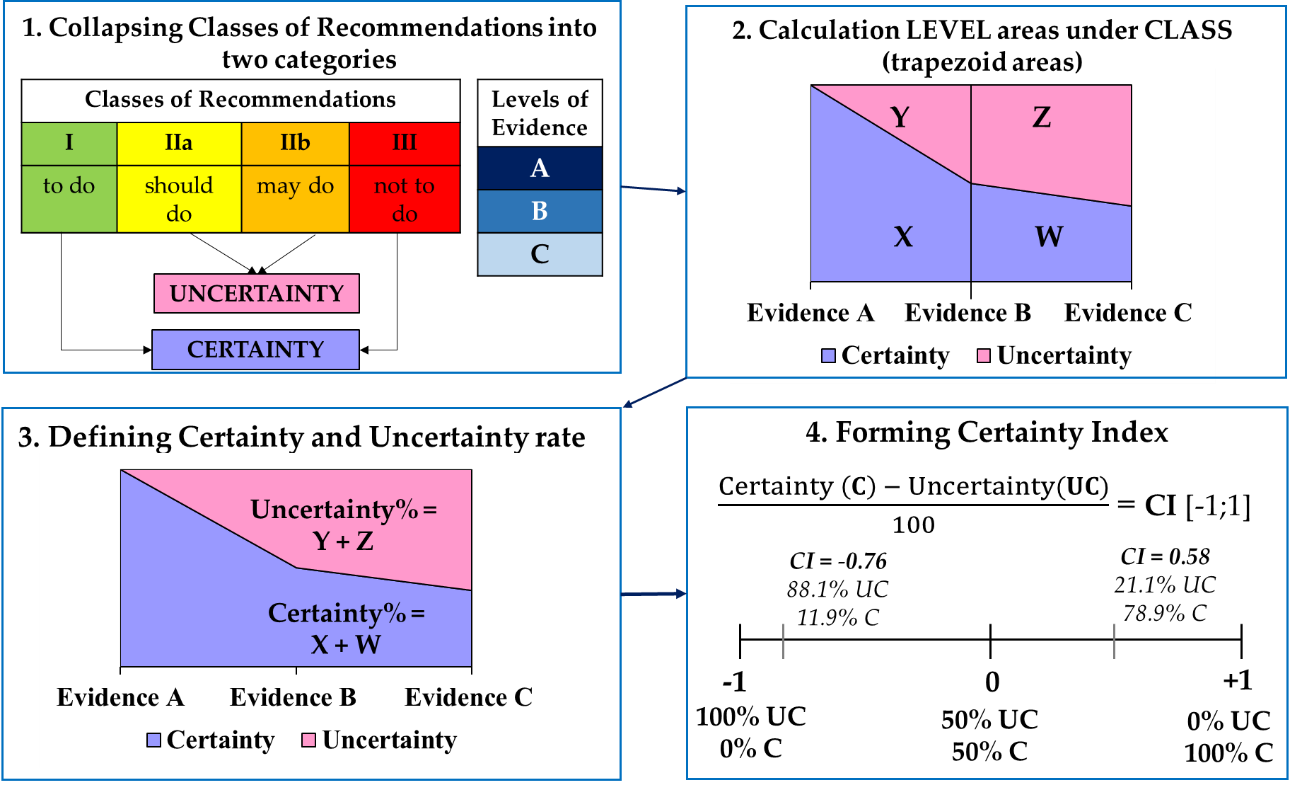
**

1. Collapsing Classes of Recommendations into two categories: to define certainty and uncertainty, we collapsed the Classes of Recommendations into two categories: uncertainty (‘should + may do’) and certainty (‘to do + not to do’). 2. Calculation LEVEL areas under CLASS: we calculated the areas using the trapezoid area formula: $=\left( a+b \right)\frac{h}{2}$, where ‘a’ and ‘b’ are the bases (parallel sides) and ‘h’ is the height of the trapezoid. 3. Defining certainty and uncertainty rate: Based on the calculation in step 2, we expressed trapezoid areas in percentages. 4. Forming certainty index: Based on the percentages in step 3, an index of certainty/uncertainty was formed which ranges from −1 to +1; a value of −1 implies 100% uncertainty, a value of +1 implies 100% certainty, and a value of 0 implies fifty–fifty certainty and uncertainty. The depicted certainty indexes (CI = −0.76 and CI = +0.58) are random examples showing the meaning of a certainty index with the rate of the percentages of certainty and uncertainty.

References:

Koller A, Takács J. A Guideline for Guidelines: A Novel Method to Assess the Helpfulness of Medical Guidelines. Journal of Clinical Medicine. 2024; 13(13):3783. https://doi.org/10.3390/jcm13133783

Koller, Akos MD, PhD1,2,3; Takács, Johanna MSc1. Translation of scientific evidence into cardiovascular guidelines. JBI Evidence Implementation 19(4):p 437-445, December 2021. | DOI: 10.1097/XEB.0000000000000266
