# Supplementary material for: Development of the European Society of Hypertension guidelines for the management of arterial hypertension: comparison of the helpfulness of ESH 2013, 2018, and 2023 guidelines
Source: J Hypertens. 2025 Feb 17;43(5):852–8. doi: 10.1097/HJH.0000000000003985 (PMC11970597; doi:10.1097/HJH.0000000000003985)
Supplement: Supplemental Digital Content [file jhype-43-852-s002.docx]

**Supplementary File 2**. Demonstration of possible conclusions based on the frequency of evidence at each Class of Recommendations (Level by Class), and the frequency of recommendations in each Level of Evidence (Class by Level)

(A) Level by Class, i.e., Evidence (%) at each Classes of Recommendations. It can be calculated the percentages of the Levels of Evidence (A, B, and C) at each Class of Recommendations.

(B) Class by Level, i.e., Recommendations (%) in each Levels of Evidence. It can also be calculated the percentages of the Classes of Recommendations (I, IIa,IIb/II, and III) at each Level of Evidence.


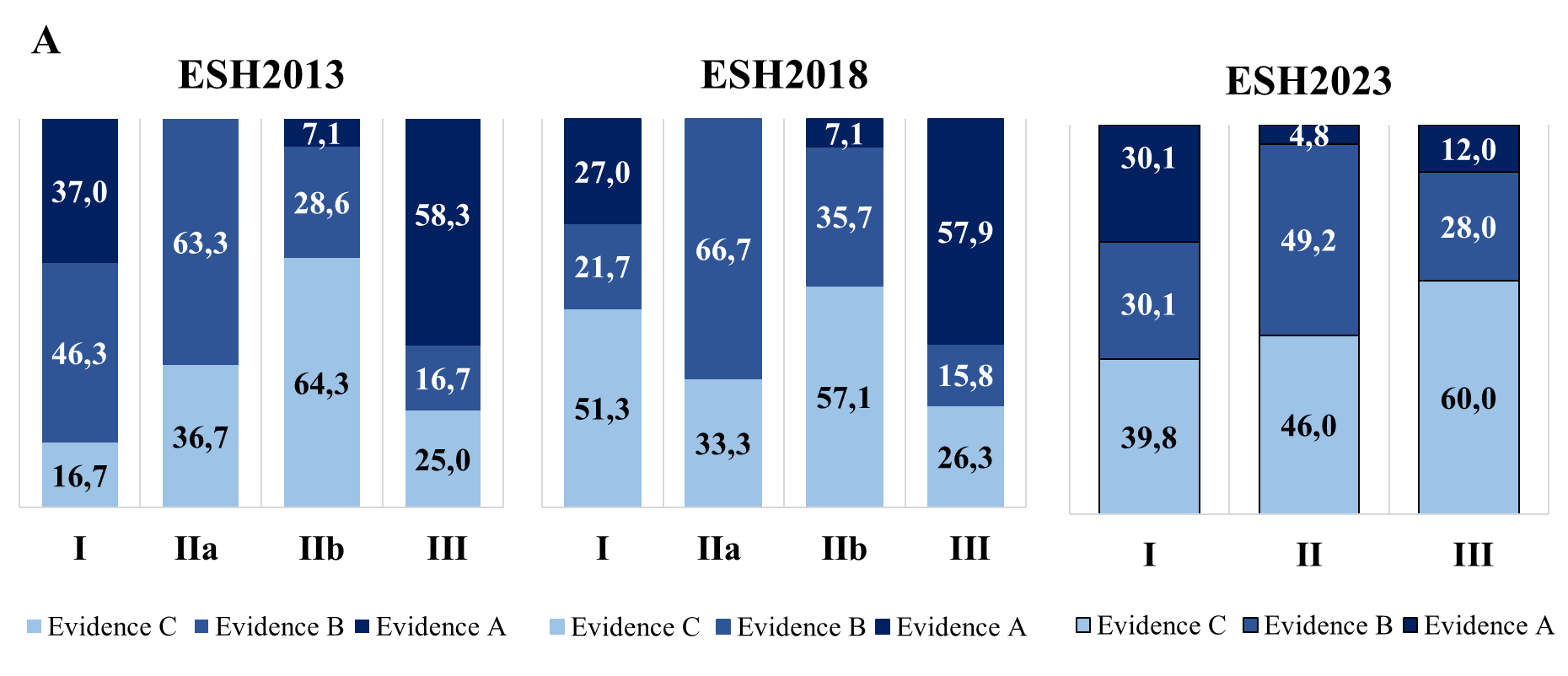


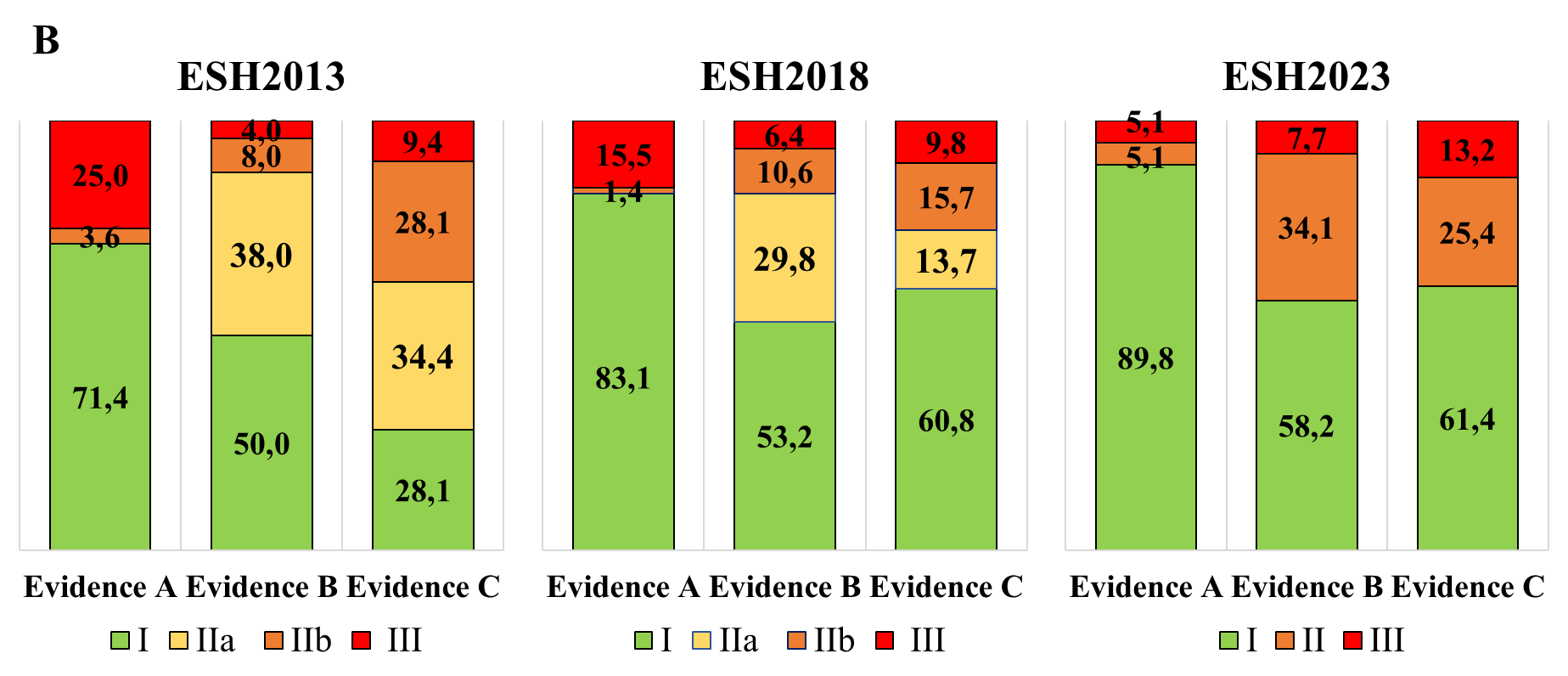


To assume a correlation between the Level of Evidence and the Classes of Recommendations is false taking into account the fact that “evidence-based guidelines”, after conducting a systematic search for evidence and evaluating its quality, provide recommendations based on the best available evidence, **even in cases when that evidence is of low quality** (GRADE Working Group, 2004). The balance between desired and unwanted outcomes, values and preferences, and costs or resource allocation determines the strength of a recommendation. Thus, a beneficial, useful, and effective recommendation can be based on evidence and/**or general agreement.** Thus, analyses based on Class by Level, and Level by Class, inevitably imply a correlation between the Level of Evidence and the Classes of Recommendations resulting in false conclusions.

References: GRADE Working Group (2004) Grading quality of evidence and strength of recommendations. BMJ 328: 1490.
